# Effects of natural blend of essential oil on growth performance, blood biochemistry, cecal morphology, and carcass quality of broiler chickens

**DOI:** 10.3382/ps.2013-03387

**Published:** 2013-12-17

**Authors:** F. Khattak, A. Ronchi, P. Castelli, N. Sparks

**Affiliations:** * Avian Science Research Centre, Scotland's Rural College (SRUC), Ayr, KA6 5HW, Scotland; † Tecnessenze S.r.l, Via Ronchi Inferiore, 32/B, 40061 Cà de Fabbri Minerbio, Bologna, Italia

**Keywords:** essential oil, broiler, growth performance, carcass quality, cecal morphology

## Abstract

The study evaluated the effect of a novel commercial preparation of natural blend of essential oils from basil, caraway, laurel, lemon, oregano, sage, tea, and thyme (Tecnaroma Herbal Mix PL) on growth performance, blood biochemistry, cecal morphology, and carcass quality of broilers. Six nutritionally adequate wheat and soybean-based diets were generated by the addition of Tecnaroma Herbal Mix PL at 0, 100, 200, 300, 400, and 500 g/t of feed. The diets were fed as crumbs in the starter phase (d 0–10) and as pellets during the grower (d 10–24) and finisher (d 24–42) phases. Nine hundred sixty 1-d-old chicks were allocated to the 6 dietary treatments each having 8 replicate pens with 20 birds per pen. The data obtained were analyzed using ANOVA with a *P* < 0.05 level of significance. Birds fed diets supplemented with Tecnaroma Herbal Mix PL had significantly heavier BW and higher (*P* < 0.05) weight gain and had improved (*P* < 0.05) feed to gain ratio compared with the control group during grower phase and overall performance. The blood biochemistry results showed no differences (*P* > 0.05) between treatments. The carcass weight, breast weight, and relative percentage of breast meat increased (*P* < 0.05) when diets were supplemented with Tecnaroma Herbal Mix PL compared with that from birds fed the control diet. The inclusion level of 300 g of Tecnaroma Herbal Mix PL/t of feed was optimum for enhancing breast meat yield and nutrient utilization as indicated by increased (*P* < 0.05) cecal villus surface area.

## INTRODUCTION

Feed additives derived from plants, including aromatic plants and essential oils (**EO**), have gained popularity since 2006 when European Union legislations phased out the use of antibiotics as feed additives in poultry feed legislation (European Probiotic Association, 2012). Essential oils have been defined as plant essences or easily evaporated benzene or terpene derivatives normally obtained from plant material by steam, water distillation, or both (Cross et al., 2007). Essential oils may exhibit a range of potentially beneficial properties including antimicrobial (Deans and Ritchie, 1987; Paster et al., 1990; Hammer et al., 1999), antioxidant (Vichi et al., 2001), antiviral (Bishop, 1995), antitoxigenic (Juglal et al., 2002), antiparasitic (Hafez and Hauck, 2006), and a digestive stimulant (Platel and Srinivasan, 2004). However, although there is a considerable body of literature providing in vitro evidence of the antibacterial, antifungal, and antiviral activity of plant extracts (Smith-Palmer et al., 1998; Lambert et al., 2001; Brenes and Roura, 2010; Lee et al., 2004; Rota et al., 2004), there are fewer in vivo studies that confirm growth-promoting effects. The present experiment was designed to assess the effectiveness of a range of concentrations of a novel commercial preparation consisting of a blend of natural herbal oils as dietary feed additive for poultry. The effects of this EO blend on growth, carcass quality, blood biochemistry, and cecal morphology were determined.

## MATERIALS AND METHODS

### Blend of EO

Tecnaroma Herbal Mix PL, a commercial feed additive containing a blend of EO from basil, caraway, laurel, lemon, oregano, sage, tea, and thyme, was used in this study. Calculated value of EO in different experimental diets is presented in Table 1. Tecnaroma Herbal Mix PL is manufactured by Tecnessenze S.r.l.

**Table 1.** Calculated and analyzed value of essential oils (EO) in experimental diets

Treatment	Calculated value of EO in feed (mg)	Analyzed value of EO in feed (mg)
1	0	0
2	15	11–13
3	30	23–27
4	45	36–41
5	60	48–54

### Bird Trial

A total of 960 male broiler chicken (Ross 308) were allocated randomly to 6 dietary treatments using a randomized complete block design. Each treatment had 8 replicate floor pens with 20 chicks per pen. Each pen measuring 1.74 m^2^ was deep littered with clean wood shaving and was equipped with nipple drinkers and hanging feeders. A nutritionally adequate wheat and soybean-based diet with no added Tecnaroma Herbal Mix PL was used as control diet (treatment 1), and its calculated composition is presented in Table 2. The other 5 treatments were generated by adding Tecnaroma Herbal Mix Pl at 100, 200, 300, 400, and 500 g/t of feed to the control diet and were designated as treatment 2, 3, 4, 5, and 6, respectively. No antimicrobial or anticoccidial products were included in the formulation. The feeding program consisted of a starter, grower, and finisher diets that were fed from d 0 to 10, 10 to 24, and 24 to 42, respectively. All 6 experimental diets were in crumbled form (starter phase) or pellets (grower and finisher phase). Chicks were reared from 1 d old on the experimental rations and were allowed ad libitum access to both feed and water throughout the study period of 42 d. The light regimen at 1 d old was 23L:1D, which was stepped down to 14L:10D by d 6, this being maintained until the end of the study. The room temperature was 32°C at the start of the trial and was reduced gradually to 21°C by 21 d of age and kept at that temperature for the remaining study duration. All chicks were vaccinated at the hatchery against infectious bronchitis. No additional vaccinations were administered during the study. Body weight and feed intake of each replicate pen was recorded at the start and end of each phase. At the end of the study at d 42, 8 birds per treatment (one bird per pen) were randomly selected and euthanized (using overdose of sodium pentobarbitone via intraperitoneal injection), and blood samples were taken in heparin tubes for blood biochemistry. After blood collection, the same birds were dissected and ceca was removed for histomorphology examination. At d 42, another 144 birds (4 birds per pen) were randomly selected, wing tagged, weighed individually, and processed for carcass quality. The birds were processed at the Carcass Evaluation Unit at SRUC. A routine commercial processing procedure was followed. All birds were euthanized using a stun bath, where DC current is set at 100 volts, 400 Hz, giving each bird a minimum of 105 mA as required. The temperature of the scald tank was set at 51.5 ± 0.3°C. Immediately after the transfer of processed birds to the chill room, all processed birds were assessed for carcass quality. The carcass data consisted of live weight, carcass weight, and breast weight (without bone) of the birds.

**Table 2.** Ingredient and nutrient compositions of the control diets (%)^1^

Item	Starter (d 0–10)	Grower (d 11–24)	Finisher (d 24–42)
Ingredient			
Wheat	65.04	65.0	65.0
Wheat feed^2^	0.0	3.18	8.6
Soyabean meal (48.5%)	27.5	22.75	16.5
l-Lysine HCl	0.42	0.35	0.35
dl-Methionine	0.29	0.25	0.25
l-Threonine	0.15	0.15	0.15
Soy oil	3.0	5.0	6.0
Limestone	0.92	0.92	0.85
Dicalcium phosphate	1.93	1.65	1.55
Salt	0.35	0.35	0.35
Vitamin mineral premix^3^	0.40	0.40	0.40
Total	100	100	100
Calculated analysis			
Ether extract	4.58	6.62	7.71
Protein	21.35	19.45	17.26
Fiber	2.84	2.95	3.19
Ash	6.21	5.81	5.54
ME (kcal/kg)	3,014	3,129	3,181
Total lysine	1.40	1.21	1.04
Available lysine	1.30	1.13	0.97
Methionine	0.57	0.51	0.47
Methionine + cysteine	0.90	0.81	0.74
Threonine	0.87	0.80	0.70
Tryptophan	0.24	0.21	0.19
Calcium	1.03	0.95	0.89
Phosphorus	0.72	0.67	0.66
Available phosphorus	0.48	0.44	0.42
Sodium chloride	0.41	0.40	0.40
Analyzed nutrient composition			
CP	22.7	20.3	19.6
Ether extract	5.6	8.5	8.8
Crude fiber	2.7	2.6	3.1

^1^The other 5 treatments were generated by adding Tecnaroma Herbal Mix Pl (Tecnessenze S.r.l.) at 100, 200, 300, 400, and 500 g/t of feed to the control diet and were designated as treatment 2, 3, 4, 5, and 6, respectively.

^2^Also known as wheat middlings, thirds, sharps, or wheatings.

^3^Vitamin mineral premix contained the following per kilogram of diet: vitamin A, 16,000 IU; vitamin D_3_, 3,000 IU; vitamin B_1_, 3 mg; vitamin B_2_, 10 mg; vitamin B_6_, 3 mg; vitamin B_12_, 15 μg; nicotinic acid, 60 mg; pantothenic acid, 14.7 mg; folic acid, 1.5 mg; iron, 20 mg; copper, 10 mg; manganese, 100 mg; cobalt, 1.0 mg; zinc, 82.2 mg; iodine, 1 mg; selenium, 0.2 mg; molybdenum, 0.5 mg. In addition, the following were added (per t): biotin, 7.5 g; choline chloride, 500 g; vitamin E, 150 g.

### Blood Biochemistry and Cecal Morphology

Blood biochemistry analysis included the measurement of alkaline phosphatase, aspartate amino transferase, alanine amino transferase, gamma-glutamyl transferase, lactate dehydrogenase, total protein, albumin, amylase cholesterol, and glucose. For cecal morphology, small sections from ceca were removed. These sections were gently flushed with PBS (pH 7.2) and then immediately fixed in 10% buffered formalin solution. The fixed intestinal sections were subsequently dehydrated by transferring through a series of ethyl alcohols with increasing concentrations (70, 80, 95, and 100%), cleared with xylene, and embedded in polyfin-embedded wax. Tissue sections (2 μm) were cut by microtome (Leitz-1512 Microtome, Leitz, Wetzlar, Germany), placed on glass slides, and stained with hematoxylin (Gill no. 2, Sigma, St. Louis, MO) and eosin (Sigma). The measurements for the villus and crypts dimension were carried out using an Olympus BX41 Laboratory Microscope (Olympus UK Ltd., Essex, UK) at ×40 and ×100 magnification. Pictures of villus and crypts were obtained with a video camera (SPOT idea, Diagnostic Instrument Inc., Sterling Heights, MI), connected with a monitor screen (Dell, Berkshire, UK), and computer, with measurements made using the Spot Basic (Diagnostic Instruments Inc., Sterling Heights, MI) imaging software. The villus height was measured from the crypt-villus junction to the brush border at the tip. Villus width was measured between brush borders of opposing epithelial cells at the midpoint of the villus where possible. Crypt widths and depths were taken at the level of the basement membranes of opposing crypt epithelial cells. Surface area was calculated using formula = (2π) × (villus width/2) × (villus height) as described by Sakamoto et al. (2000).

### Statistical Analysis

The study design was a randomized complete block design with 6 blocks and 6 treatments. The data obtained were subjected to ANOVA using a GenStat 14 statistical software package (IACR, Rothamstead, Hertfordshire, UK). Experimental unit was a pen. Pairwise contrasts were used to show differences between the treatments.

The study was subject to ethical review and approved by the Animal Experiments Committee of SRUC.

## RESULTS

Analyzed EO, CP, ether extract, and crude fiber values were within the expected range (Tables 1 and 2). The growth performance data during starter (0 to 10 d), grower (10 to 24 d), and finisher (24 to 42 d) phases are presented in Table 3. There were no treatment differences (*P* < 0.05) in growth performance response during starter phase. During the grower phase, all the birds fed Tecnaroma Herbal Mix PL had heavier (*P* < 0.05) BW and better weight gains compared with the birds on the control treatment. This increase in BW was also reflected in the improved (*P* < 0.05) feed to gain ratios for birds fed Tecnaroma Herbal Mix PL compared with the control group. During the finisher phase, the birds in treatment 2, 3, and 6 were heavier (*P* < 0.05) in BW compared with 1 (control group). The overall data (d 0 to 42) showed significantly higher BW gains and lower (*P* < 0.05) feed to gain ratio for the birds fed Tecnaroma Herbal Mix PL compared with control group. No differences were observed when average feed intake data were compared between the treatments during the different phases of growth. The birds remained healthy throughout the trial period, and the percentage mortality for treatment 1 to 6 was 7.8, 5.0, 3.8, 6.9, 5.0, and 2.5%, respectively. Mortality was not associated with treatment (*P* > 0.05).

**Table 3.** Effect of experimental diets on growth performance of broilers^1^

	Starter phase (d 0–10)			Grower phase (d 10–24)			Finisher phase (d 24–42)			Overall performance (d 0–42)		
Item	Gain (kg/bird)	FI^2^ (kg/bird)	F:G^3^ (kg:kg)	Gain (kg/bird)	FI (kg/bird)	F:G (kg:kg)	Gain (kg/bird)	FI (kg/bird)	F:G (kg:kg)	Gain (kg/bird)	FI (kg/bird)	F:G (kg:kg)
Treatment												
1	0.272	0.319	1.175	1.012	1.533	1.518	1.913	3.873	2.035	3.197	5.725	1.79
2	0.267	0.316	1.178	1.087	1.519	1.402	2.073	3.900	1.903	3.427	5.735	1.683
3	0.267	0.309	1.152	1.080	1.507	1.402	2.087	3.885	1.868	3.435	5.701	1.661
4	0.265	0.308	1.16	1.082	1.515	1.404	2.029	3.791	1.902	3.376	5.613	1.675
5	0.264	0.313	1.168	1.085	1.522	1.411	2.039	3.885	1.926	3.389	5.72	1.695
6	0.266	0.314	1.193	1.066	1.51	1.417	2.086	3.784	1.825	3.418	5.609	1.645
SEM	0.001	0.002	0.008	0.006	0.006	0.012	0.021	0.022	0.023	0.022	0.026	0.013
*P*-value for contrasts^4^												
1 versus 2	0.331	0.689	0.934	0.001	0.507	0.004	0.031	0.687	0.075	0.002	0.902	0.013
1 versus 3	0.362	0.201	0.433	0.003	0.211	0.004	0.019	0.858	0.027	0.002	0.761	0.003
1 versus 4	0.196	0.163	0.602	0.002	0.383	0.004	0.110	0.213	0.074	0.015	0.166	0.008
1 versus 5	0.171	0.463	0.797	0.002	0.598	0.007	0.084	0.856	0.141	0.009	0.953	0.026
1 versus 6	0.236	0.575	0.534	0.015	0.263	0.010	0.020	0.181	0.006	0.003	0.149	0.001

^1^All means are average of 8 pens per treatment.

^2^FI = feed intake.

^3^F:G = feed-to-gain ratio.

^4^Significance level (*P* ≤ 0.05).

The carcass data obtained are presented in Table 4. The live weight of the birds processed showed no differences (*P* > 0.05) between the treatments. The carcasses were heavier (*P* < 0.05) for birds in treatment 4, 5, and 6 compared with 1 (control). Heaviest breast weights (*P* < 0.05) were recorded for birds in treatments 4 and 5, which were 10.4 and 7.4%, respectively, heavier than treatment 1. Similar trend was noted in percentage breast data, where birds in treatment 4 and 5 had higher values (*P* < 0.05) compared with treatment 1.

**Table 4.** Effect of experimental diets on carcass quality of broilers^1^

Item	Live weight^2^ (g)	Carcass weight (g)	Breast weight^3^ (g)	Carcass^4^ (%)	Breast^5^ (%)
Treatment						
1	3,212	2,282	649.3	71.59	28.3
2	3,287	2,336	672.6	70.14	28.8
3	3,236	2,339	677.8	71.87	28.9
4	3,341	2,383	716.9	70.83	30.1
5	3,301	2,398	697.9	71.39	29.6
6	3,319	2,377	684.3	71.02	28.9
SEM	20.600	13.450	5.920	0.200	0.156
*P*-value for contrasts^6^						
1 versus 2	0.290	0.210	0.213	0.027	0.306
1 versus 3	0.738	0.187	0.129	0.665	0.183
1 versus 4	0.068	0.020	<0.001	0.239	<0.001
1 versus 5	0.206	0.008	0.010	0.755	0.012
1 versus 6	0.130	0.029	0.062	0.375	0.215

^1^All means are average of 24 birds per treatment.

^2^Live weight used here is of the birds used in processed data.

^3^Breast was boneless.

^4^% carcass = (carcass weight/live weight × 100).

^5^% breast = (breast weight/carcass weight × 100).

^6^Significance level (*P* ≤ 0.05).

Histomorphology data for the ceca are presented in Table 5. Villus height was higher (*P* < 0.05) for birds in treatment 2 compared with those in treatment 1, 3, 4, and 6. Whereas birds in treatment 3, 4, and 6 had higher (*P* < 0.05) values for villus width compared with treatment 1. This increase in villus measurements was also reflected in the increase (*P* < 0.05) in the villus surface area of birds in treatment 4 and 6 compared with treatment 1.

**Table 5.** Effect of experimental diets on the cecal morphology of 42-d-old broilers^1^

Item	Villus height (μm)	Villus width (μm)	Crypt depth (μm)	Crypt width (μm)	Surface area (μm^2^)
Treatment					
1	291	102	101	82	94,766
2	320	95	98	78	95,496
3	286	114	97	76	104,010
4	291	122	98	78	111,196
5	309	103	101	77	98,974
6	299	118	99	81	110,552
SEM	3.037	1.711	0.992	0.772	1,741
*P*-value for contrasts^2^					
1 versus 2	0.005	0.190	0.358	0.195	0.896
1 versus 3	0.569	0.024	0.269	0.020	0.097
1 versus 4	0.950	<0.001	0.355	0.161	0.003
1 versus 5	0.078	0.913	0.925	0.055	0.449
1 versus 6	0.451	0.004	0.548	0.693	0.005

^1^Mean represents 4 birds per treatment and the average of 10 measurements per parameter per bird.

^2^Significance level (*P* ≤ 0.05).

There were no differences (*P* > 0.05) between treatments in blood biochemistry parameters except for lactate dehydrogenase values, which were lower (*P* < 0.05) for birds in treatments 2 and 3 (1,652 and 1,561 IU/L) compared with those in treatments 1 and 6 (2,056 and 2,213 IU/L). However, all values were within the expected physiological range (860 to 2,900 IU/L).

## DISCUSSION

Inclusion of EO in poultry feed did not improve growth performance during the starter phase. This could be due to digestive enzyme secretion capacity, which is reported to be relatively low in young chicks, only increasing toward d 21 (Noy and Sklan, 1995). Improved growth performance during the grower and finisher phase could be attributed to the presence of EO in diet, which encourages secretions of endogenous digestive enzymes, which then enhance nutrient digestion and gut passage rate in chickens (Lee et al., 2003, 2004). Similar results were reported by Amad et al. (2011) who reported that phytogenic feed additives improved apparent ileal digestibility of nutrients at 21, 35, and 42 d of age. Synergistic effects on growth rates of feeding oil combinations have also been reported (Bassett, 2000; Langhout, 2000; Alcicek et al., 2003; Denli et al., 2004). However, in contrast, there are studies where the effects on animal performance were nonsignificant (Case et al., 1995; Botsoglou et al., 2002). The contributory factors causing these differences reported in other studies could be attributed to the differences in the inclusion levels of EO, sources of herbs used to form blend of EO, basal diet composition, or the microbial environment in which the birds were reared.

Blood biochemistry profiles were within the expected range, and no signs of toxicity were observed. This would be predicted based on the rapid metabolic conversion and excretion of EO from the body and are consistent with those reported by Hagan et al. (1967) who found no clear signs of toxicity when rats were fed diets containing thymol at the level of 1,000 and 10,000 mg/kg for 19 wk. It has been suggested that birds fed EO have reduced concentrations of serum cholesterol and that the hypocholesterolemic effect of EO is due to compounds in EO that have the ability to inhibit hepatic 3-hydroxy-3-methylglutaryl coenzyme A reductase activity, a key regulatory enzyme in cholesterol synthesis (Yu et al., 1994; Case et al., 1995). However, in the current study, no differences were observed when cholesterol values were compared between the treatments. This nonsignificant effect could be associated with the selection of EO components used in Tecnaroma Herbal Mix PL, which were either ineffective in inhibiting hepatic 3-hydroxy-3-methylglutaryl coenzyme A reductase or due to their fast degradation rate in the liver.

Dietary supplementation of Tecnaroma Herbal Mix PL at 300 g/t of diet in this study significantly increased villus width and surface area, indicative of improved nutrient absorption (Geyra et al., 2001) and performance (Choct, 2009). The increase in villus surface area may be the explanation for why birds fed 300 g/t had higher breast yield. The effects of Tecnaroma Herbal Mix PL on the intestinal microflora were not evaluated in this study; however, others have shown that EO have the capacity, when fed to broilers, to reduce the growth of *E. coli* and *C. perfringens* (Losa and Kohler, 2001) and increase the numbers of *Lactobacillus*spp. (Tucker, 2002). Similarly, Cross et al. (2007) found that the inclusion of thyme, marjoram, and rosemary in broiler diets reduced the numbers of cecal *C. perfringens* by >1 log.

In conclusion, data from this study showed that the addition of at least 100 g/t of Tecnaroma Herbal Mix PL in a diet meeting the nutrient requirements of broilers would improve the BW and weight gains with positive effects on feed to gain ratio. The improvement in growth performance was not dose dependent; no additional benefits were identified when the Tecnaroma Herbal Mix PL was added at concentrations greater than 300 g/t.
